# Insulin Modulates NK Cell Activity in Liver Fibrosis MASH Patients via the STING Pathway

**DOI:** 10.3390/cells14130941

**Published:** 2025-06-20

**Authors:** Johnny Amer, Ahmad Salhab, Amiram Ariel, Rifaat Safadi

**Affiliations:** 1 The Liver Institute, Hadassah Medical Hospital, Jerusalem 91120, Israel; ahmad.salhab@mail.huji.ac.il (A.S.); safadi@hadassah.org.il (R.S.); 2 The Department of Human Biology, University of Haifa, Haifa 3498838, Israel; amiram@research.haifa.ac.il

**Keywords:** liver fibrosis, NK cells, STING, MASH, insulin receptor

## Abstract

**Background:** The STING (Stimulator of Interferon Genes) pathway plays a vital role in the body’s innate immune defense system, primarily involved in DNA sensing and type I interferon production. While STING is well-established in various immune cells, its role in natural killer (NK) cells, particularly within the context of liver fibrosis, remains inadequately explored. **Aim:** The current study investigates the relationship between STING expression, NK cell activity, and insulin receptor (IR) signaling in patients with metabolic dysfunction-associated steatohepatitis (MASH). **Methods:** Peripheral NK cells were isolated from healthy controls and MASH patients with varying stages of liver fibrosis (early: F1/F2; advanced: F3/F4). The expressions of STING, IR, NK cell activation markers (CD107a, NKp46), and NK cell inhibitory markers (LAIR-1, Siglec-7) were assessed using flow cytometry. NK cell cytotoxicity against primary hepatic stellate cells (pHSCs) was evaluated through apoptosis assays. STING agonists (2′3′-cGAMP and DMXAA) were used to stimulate NK cells, and their effects on STING expression, NK cell activation, and cytotoxicity were measured. Additionally, the impact of insulin signaling on STING expression and NK cell function was examined. **Results:** Our results demonstrate that STING expression in NK cells correlates with disease severity in liver fibrosis. NK cells from MASH patients with advanced fibrosis (F3/F4) showed inhibited STING protein levels that were statistically comparable to healthy NK cells and accompanied by impaired cytotoxicity and decreased IFN-γ production. In contrast, NK cells from early fibrosis (F1/F2) exhibited higher STING expression and better functional activity. STING agonist treatment (2′3′-cGAMP) restored STING expression and enhanced NK cell activity across all fibrosis stages. Furthermore, insulin treatment and combined insulin and 2′3′-cGAMP treatment synergistically upregulated both IR and STING expressions, leading to improved NK cell function and increased cytotoxicity, particularly in advanced fibrosis. **Conclusion:** Our results highlight the potential of targeting STING and insulin signaling pathways as a therapeutic approach in restoring NK cell function and enhance immune surveillance in liver fibrosis.

## 1. Introduction

Metabolic dysfunction-associated steatohepatitis (MASH), the progressive inflammatory form of metabolic-associated fatty liver disease (MAFLD), has become one of the leading causes of chronic liver disease worldwide [1,2]. In MASH, however, chronic metabolic inflammation impairs immune surveillance mechanisms, particularly those mediated by natural killer (NK) cells and macrophages, leading to unchecked activation of hepatic stellate cells and fibrogenesis [3,4,5,6]. One of the central innate immune pathways implicated in liver immunoregulation is the Stimulator of Interferon Genes (STING) pathway [7]. Acting as a cytosolic DNA sensor, STING activates type I interferon production via the cyclic GMP-AMP synthase (cGAS)-STING axis [8,9]. STING is shown to be expressed in innate immune cells, including macrophages, dendritic cells (DCs), and monocytes, and is thought to regulate their functions through the induction of pro-inflammatory cytokines and enhancement of immune cell recruitment [10,11,12]. cGAS-STING signaling has demonstrated an expanding role in microbial infections, cellular senescence, autoimmune and inflammatory diseases, and antitumor immunity [13,14]. DNA or cGAMP from tumors activates cGAS-STING signaling to initiate antitumor immunity [15]. Macrophage STING signaling has been shown to promote NK cells suppression of colorectal cancer liver metastasis via 4-1BBL/4-1BB co-stimulation [16]. Lu et al. demonstrate that tumor-derived cGAMP transfer and subsequent cell intrinsic STING engagement prime NK cell antitumor response [17]. They identify the previously unrecognized role of STING signaling in maintaining a reservoir of TCF-1 + NK cells in tumors [17]. STING agonism represents a promising therapeutic strategy to enhance NK cell-based cancer immunotherapy [17,18,19].

We have previously demonstrated the crucial antifibrotic effects of NK cells in killing activated hepatic stellate cells and delaying liver fibrogenesis [20,21]. Amer et al. investigated the potential role of insulin resistance through the expression of insulin receptors as a metabolic immune checkpoint leading to NK impairment and eventual MASH progression and suggested a new cellular insulin checkpoint through which NK cells contribute to fibrosis in patients with MAFLD [22].

Despite STING’s established immunomodulatory functions, its expression and potential role in regulating NK cell activity within the fibrotic liver microenvironment remain unclear. Furthermore, the possible crosstalk between STING and IR signaling pathways in modulating NK cell effector functions remains unexplored. The present study investigates whether dysregulation of peripheral NK cell activity in advanced liver fibrosis is associated with alterations in STING and IR expression. Unraveling this relationship may offer novel insights into the immunometabolic mechanisms underlying liver fibrosis progression and identify potential therapeutic targets.

## 2. Materials and Methods

### 2.1. Patients and Healthy Donors

Adult patients (aged ≥18 years) with histologically diagnosed MASH were enrolled at the Hadassah Medical Hospital between 2018 and 2025. Patient demographic and clinical characteristics are presented in Table 1. Healthy volunteers without MASH [×10] or liver fibrosis [*n* = 20], as evident by biopsy assessments and confirmed by FibroScan elastography (a tool for determining the degree of liver fibrosis via measuring stiffness). Patients who had a history of immunosuppressant administration and who had used specific medications (warfarin, metformin, glitazones, glucagon-like peptide-1 receptor agonists, glucose-dependent insulinotropic polypeptide agonists, sodium–glucose cotransporter-2 inhibitors and insulin) within the two months before sampling; who had any active or past malignancy; or who had excessive daily alcohol intake (>20 gm/day for women and > 25 gm/day for men) were excluded. Patients with non-MASH liver diseases, including viral hepatitis, autoimmune liver disease, hemochromatosis, Wilson’s disease, alpha-1-antitrypsin disease, or other toxicities, were also excluded from this study.

The degree of liver fibrosis was evaluated in the included patients by a single specialized pathologist using METAVIR (scores: F0, no fibrosis; F1, perisinusoidal or periportal fibrosis; F2, perisinusoidal and periportal fibrosis; F3, bridging fibrosis with architectural distortion; and F4, cirrhosis). Due to the limitations associated with distinguishing METAVIR stages, we combined F1 and F2 scores as “early liver fibrosis” and those with F3 and F4 scores as “advanced liver fibrosis”.

This study was approved by the Ethics Committees of the Hadassah Medical Organization (HMO; approvals HMO-0605-16 and HMO-57398-AR). Written informed consent was obtained from all participants. All procedures involving human subjects were conducted in accordance with the ethical standards outlined in the Declaration of Helsinki.

### 2.2. Peripheral NK Cell Isolation

Peripheral blood (20 mL) was collected by venipuncture, and each sample was transferred to a 5 mL sodium citrate tube. Blood samples were diluted with an equal volume of phosphate-buffered saline (PBS; Merck, Jerusalem, Israel), mixed gently, and layered on top of an equal volume of Ficoll-Paque (Cat# GE17-1440-02; Merck, Jerusalem, Israel) in a separate tube. The tube was centrifuged at 515× *g* (with gentle acceleration and no braking) for 20 min at room temperature. The lymphocyte-rich middle layer was carefully collected under sterile conditions and transferred to a fresh tube. The cells were washed with PBS or a cell wash buffer, gently resuspended, and centrifuged at 515× *g* for 10 min to eliminate any remaining contaminants. NK cells were then isolated from the lymphocyte population using the Human NK Cell Isolation Kit (Cat# 17955; STEMCELL, Tel Aviv, Israel), which employs a negative selection strategy in accordance with the manufacturer’s protocol. In this process, non-NK cells including T cells, B cells, stem cells, dendritic cells, monocytes, granulocytes, and erythroid cells were selectively labeled with a biotin-conjugated antibody cocktail (NK Cell Rapid Spheres Cocktail, STEMCELL, Tel Aviv, Israel) and removed using magnetic separation, resulting in a purified population of NK cells.

### 2.3. Isolation of Primary Hepatic Stellate Cells (pHSCs) from Liver Biopsies

Isolated pHSCs from liver tissue samples were obtained from patients with advanced liver fibrosis due to MASH. The tissue was enzymatically digested at 37 °C for 40 min in 20 mL of Gey’s Balanced Salt Solution containing 0.1% (wt/vol) pronase, 0.1% (wt/vol) collagenase, and 0.01% (wt/vol) deoxyribonuclease I (DNase I) (Roche Diagnostics GmbH, Mannheim, Germany). Cell viability was assessed by flow cytometry (see below), with isolated pHSCs consistently showing viability greater than 90%, confirming their integrity and suitability for downstream applications.

### 2.4. NK Cell Cytotoxicity Assay

To assess NK cell cytotoxicity, pHSCs (1 × 10^6^ cells/mL) isolated from MASH patients with advanced liver fibrosis were cocultured at a 1:1 ratio with peripheral NK cells derived from healthy donors and patients with early or advanced liver fibrosis. Cytotoxic activity was evaluated by measuring pHSCs apoptosis via flow cytometry. All cells were maintained in Dulbecco’s Modified Eagle Medium (DMEM, Biological industry, Galilee, Israel) supplemented with 10% (vol/vol) fetal calf serum (FCS, Biological industry, Galilee, Israel) to simulate an activated environment, along with 100 units/mL penicillin and 100 mg/mL streptomycin. Cultures were incubated at 37 °C in a humidified atmosphere with 5% CO_2_. Mycoplasma contamination was ruled out using the EZ-PCR mycoplasma detection kit (Cat# 20-700-20, Biological industry, Galilee, Israel).

### 2.5. NK Cell Treatment

Following isolation, primary NK cells were seeded at 1×10^6^ cells/mL and treated for 24 h with the STING agonists 5,6-dimethylxanthenone-4-acetic acid (DMXAA; D5817, MERCK, Jerusalem, Israel) or 2′3′-cyclic GMP-AMP (2′3′-cGAMP; tlrl-nacga23-02, InvivoGen, San Diego, CA, USA), each at a final concentration of 10 µM. Recombinant human insulin (91077C, MERCK, Jerusalem, Israel) was also administered at 10 nM DMSO is used as a vehicle.

### 2.6. Flow Cytometry

All antibodies were incubated with the isolated cell suspensions (1:100) at 4 °C for 45 min. The cells were washed twice with PBS supplemented with 1% FCS, and a secondary antibody (1:100) was added for 45 min at 4 °C.

The primary human antibodies used were anti-CD45 (PE-CY-7; ab239317, Abcam, Cambridge, UK), anti-CD3 (PE; ab243075, Abcam, Cambridge, UK), anti-CD56 (PERCP-CY-5; ab206632, Abcam, Cambridge, UK), anti-CD107a (lysosomal-associated membrane protein-1 (LAMP-1) (FITC; ab187591, Abcam, Cambridge, UK), anti-NKp46 (Unconjugated; ab244208, Abcam, Cambridge, UK), anti-LAIR-1 (APC; ab270642, Abcam, Cambridge, UK), anti-Slglec (FITC; 130-132-478, Miltenyi Biotec, Bergisch, Germany), anti-IFN-γ (AF594; ab317059, Abcam, Cambridge, UK), anti-STING (AF594; ab207288, Abcam, Cambridge, UK), anti-IR (Unconjugated; ab983, Abcam, Cambridge, UK), anti-PD-1 (Unconjugated; ab52587, Abcam, Cambridge, UK), anti-TIGIT (Unconjugated; ab321793, Abcam, Cambridge, UK), and anti-LAG-3 (Unconjugated; ab209236, Abcam, Cambridge, UK). The secondary antibodies used were goat-anti-mouse IgG (AF488) (ab150113, Abcam, Cambridge, UK), goat-anti-rabbit IgG (APC) (A10931, Thermo Fisher, Kiryat Shmona, Israel). Isotype IgG labeled with the relevant fluorochrome was used as a control for each antibody.

Prior to flow cytometry analysis, the viability of pHSCs and NK cells (100 µL at 1 × 10^6^ cells/mL) was assessed using propidium iodide (PI) staining, following the manufacturer’s protocol (A35110, R&D Systems, Minneapolis, MN, USA). Samples with ≥90% PI-negative cells were considered viable. Apoptosis was determined using annexin V staining (A35110, R&D Systems, Minneapolis, MN, USA). Early apoptotic cells were identified as annexin V^+^/PI^−^, while late apoptotic cells were defined as annexin V^+^/PI^+^. The Gating strategy was set to identify the isolated NK cells following the Human NK Cell Isolation Kit and their purity was defined as CD56^+^ CD3^−^ cells to effectively exclude NKT cells from the analysis (Figure S1). All stained samples were analyzed on a BD LSR Fortessa Cell Analyzer (Becton Dickinson, Immunofluorometry Systems, Caesarea, Israel), and data were processed using FCS Express 7 software (De Novo Software, Jerusalem, Israel) for flow cytometry analysis.

### 2.7. RNA Isolation, cDNA Preparation, and Real-Time PCR

Total cellular RNA (2 μg/μL, 98% purity, determined using a NanoDrop ND-1000 spectrophotometer, NanoDrop Technologies, Jerusalem, Israel) was isolated from human peripheral blood NK cells from healthy individuals, and MASH patients with different fibrosis scores using 2 mL of TRI reagent (Cat# 90102331; Bio Lab, Jerusalem, Israel). The samples were centrifuged (1400 rpm) for 15 min at 4 °C, after which the RNA-containing supernatants were collected. For RNA precipitation, the supernatants were transferred to a new microcentrifuge tube, 0.5 mL of isopropanol (Cat# 16260521; Bio Lab, Jerusalem, Israel) was added, the samples were incubated at 25 °C for 10 min, and centrifuged (12,000 rpm) for 10 min at 4 °C. The supernatants were removed, and 1 mL of 75% ethanol was added to the pellets before centrifugation (7500 rpm) for 5 min. The pellets were air-dried at room temperature for 15 min, and then 50 μL of DEPC-treated water was added. The samples were incubated for 10 min at 55 °C. cDNA was prepared with a High-Capacity cDNA Isolation Kit (Cat# 1406197; R&D; Minneapolis, MN, USA). Real-time PCR was performed to quantify STING gene expression using TaqMan Fast Advanced Master Mix (Cat# 4371130, Applied Biosystems, Foster City, CA, USA). Gene expression was normalized to that of the housekeeping gene GAPDH. The cycling conditions for the one-step RT–PCR involved 40 cycles of 94 °C for 30 s, 60 °C for 30 s, and 72 °C for 1 min, followed by a final extension at 72 °C for 10 min. The data were analyzed using a QuantStudio™ 5 Real-Time PCR System (Cat# A34322, Applied Biosystems, Foster City, CA, USA).

### 2.8. Statistical Analysis

Statistical differences were analyzed with two-way ANOVA with Newman–Keuls post-tests among multiple groups) in Graph Pad Prism 5.0 (Graph Pad Software, Jerusalem, Israel).

## 3. Results

### 3.1. NK Cells Activity from MASH Patients Is Inversely Correlated with STING Expression in Advanced Liver Fibrosis and Influences Their Ability to Kill pHSCs

**Figure 1:**
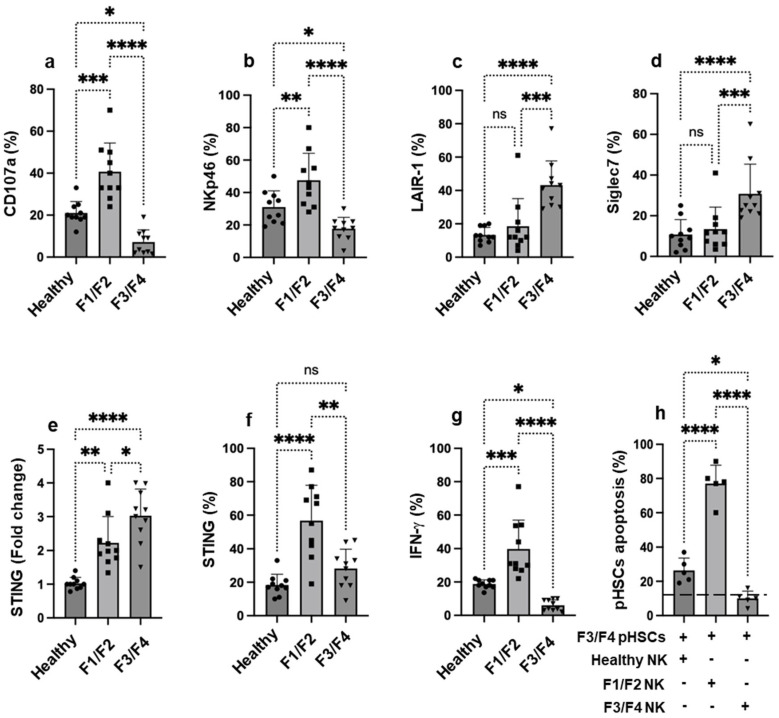
NK cell dysfunction is linked to suppressed STING expression in MASH patients with advanced liver fibrosis. The percentages of cells expressing (**a**) CD107a, (**b**) NKp46, (**c**) LAIR-1, and (**d**) Siglec7 in NK from healthy controls and metabolic dysfunction-associated steatohepatitis (MASH) patients with early (F1/F2) and advanced (F3/F4) fibrosis scores as determined by METAVIR were measured by flow cytometry. (**e**) Quantitation of *STING* mRNA in peripheral NK cells obtained from healthy individuals (considered as control) and patients with MASH with varying degrees of fibrosis scoring were assessed via RT-PCR. The data are presented as a fold change compared with healthy controls. The housekeeping gene *GAPDH* was used as the internal control for normalization. Flow cytometry analysis showed the percentages of cells expressing (**f**) STING and (**g**) IFN-γ in NK from healthy controls and MASH patients with early (F1/F2) and advanced (F3/F4) fibrosis. (**h**) A coculture cytotoxicity assay using a 1:1 ratio of NK cells (from healthy donors and MASH patients with early and advanced fibrosis scores) and pHSCs obtained from F3/F4 MASH patients was performed, and annexin V (%) expression was assessed in the pHSCs as a marker of apoptosis using flow cytometry. The horizontal dotted line indicates the baseline results from monoculture conditions. In the flow cytometry experiments, an isotype-matched IgG antibody conjugated to the appropriate fluorochrome was used as a control. Data are expressed as the mean ± standard deviation (SD). Statistical significance was assessed using Newman–Keuls two-way analysis of variance (ANOVA), with significance levels denoted as follows: * *p* < 0.01, ** *p* < 0.001, *** *p* < 0.0001, and **** *p* < 0.00001. ns = not significant. Group sizes were as follows: healthy controls (*n* = 10), early liver fibrosis (*n* = 10), and advanced liver fibrosis (*n* = 10).

To investigate the correlation between NK cell functional status and STING expression across different stages of liver fibrosis, peripheral NK cells were isolated as described in Section 2. Figure 1a shows that the tested degranulation marker of surface CD107a expression significantly declined in NK cells from MASH patients with advanced-stage fibrosis (F3/F4) of 10.4% ± 2.0 as compared to 20.3% ± 4.8 in healthy controls, with a 2-fold decrease (*p* < 0.01). In contrast, NK cells from MASH patients with F1/F2 (early-stage fibrosis) exhibited significantly elevated levels of CD107a expression with a 2.1-fold increase as compared to their healthy counterparts (*p* < 0.0001). Additional activatory and inhibitory NK cell markers were tested to confirm this phenomenon. Figure 1b demonstrated NKp46 activating receptor showed to be significantly downregulated in F3/F4 patients (17.5% ± 5.1) compared to healthy individuals (30.3% ± 10.3, *p* < 0.01), while NK cells from MASH patients with F1/F2 scores showed an expression of 45.2% ± 15.2 and was statistically significantly compared to healthy controls (*p* < 0.001).

We further characterize NK cells inhibitory checkpoint receptors. Figure 1c showed LAIR-1 expression of 15.4% ± 4.1 in healthy controls with comparable expressions of 23.1% ± 5.2 in NK cells obtained from F1/F2 (*p* = ns). NK cells from F3/F4 severity demonstrated elevated expressions of LAIR-1 with 43.8% ± 16.3, which were statistically significant (*p* < 0.00001). In parallel, the same analysis were performed using mean fluorescence intensities (MFI) for NKp46 and LAIR-1 confirmed same pattern of results (Figure S2). Furthermore, siglec7, an additional inhibitory marker, displayed an increase in its expression in F3/F4 NK cells of 2 fold as compared to both healthy and F1/F2 cells (Figure 1d, *p* < 0.00001), indicating similar patterns of results obtained with the LAIR-1 expression. Overall data indicate impaired NK cell activity via decreased activatory and increased inhibitory receptors along disease severities.

In addition, analysis of peripheral NK cells revealed a progressive increase in the expression of NK exhaustion markers of PD-1, TIGIT, and LAG-3 with advancing stages of liver fibrosis (Table 2). In healthy donors, baseline expression levels of PD-1, TIGIT, and LAG-3 were 8 ± 2.5%, 13 ± 1.4%, and 9 ± 1.5%, respectively. In patients with early-stage fibrosis (F1/F2), PD-1, TIGIT, and LAG-3 remained relatively unchanged (11 ± 3.3%, 15.3 ± 2%, and 8 ± 1.5%), respectively. However, in patients with advanced fibrosis (F3/F4), a significant elevation was observed across all markers: PD-1 (32 ± 4.7%), TIGIT (28 ± 5.3%), and LAG-3 (21 ± 4.9%) (*p* < 0.05 vs. healthy controls). These findings indicate a state of progressive NK cell exhaustion or dysfunction in advanced fibrosis, potentially contributing to impaired immune surveillance and sustained fibrogenesis in MASH.

Given the emerging role of STING in immune regulation and the limited data linking its expression to liver fibrosis and associated NK cell modulation, we evaluated STING expression at both the mRNA and protein levels. Figure 1e shows data of mRNA expressions of STING using the RT-PCR demonstrating a linear positive association with liver fibrosis severities of 2.5 and 3.0 fold in F1/F2 and F3/F4 in MASH patients, respectively, as compared to healthy NK cells (*p* < 0.001). Moreover, Figure 1f displays flow cytometry analysis of STING expression, indicating a 19.7% ± 4.2 in NK cells of healthy controls, while elevated expressions of 58.5% ± 16.7 were obtained in the F1/F2 NK cells counterparts. Interestingly, NK cells from MASH patients with F3/F4 did show the expected increase in STING expression, and data showed an inhibited STING expression of 25.1%± 1.2, that were statistically comparable to healthy NK cells (*p* = ns).

The observed discrepancy between STING mRNA upregulation and reduced protein levels in F3/F4 MASH patients suggests a post-transcriptional or translational regulation, possibly leading to functional impairment of NK cells in severe disease.

Altered STING expression in NK cells during MASH progression modulates IFN-γ production, with potential implications for NK cell-mediated antifibrotic immunity. Evaluating IFN-γ alongside STING is thought to uncover functional shifts in NK cell activity across fibrosis stages and support therapeutic targeting strategies [10,11,12]. Figure 1g revealed a marked decrease in IFN-γ expressions in the F3/F4-scored NK cells of 8.1% ± 5.2 as compared to healthy controls expressing 19.8% ± 3.7 levels (*p* < 0.01), while significantly higher IFN-γ expressions were observed in F1/F2 with 40.5% ± 17.6 (*p* < 0.0001). Our data showed that reduced STING protein in the F3/F4-scored NK cells is accompanied by reduced IFN-γ production, reinforcing the notion of impaired STING-mediated NK cell functionality in advanced disease.

To further assess functional cytotoxicity related to STING expression, peripheral NK cells from healthy and MASH patients were co-cultured with liver pHSCs obtained from F3/F4 of MASH patients. Figure 1h illustrates a high apoptosis rate of pHSCs followed by co-cultures with NK cells from healthy and F1/F2, reaching 25.2% ± 6.6 and 79.4% ± 4.3, respectively, as compared to the Basel apoptosis rate of 12.5% ± 3.8 of monocultured pHSCs (doted horizontal line, *p* < 0.0001). In contrast, co-cultures with NK cells from F3/F4 patients did not significantly change the apoptosis rate of pHSCs and remained within 12.5% ± 3.8 of those from F1/F2 (79.4% ± 4.3) and healthy donors (*p* < 0.01).

Altogether, our data suggest that STING expression positively correlates with NK cell cytotoxicity against pHSCs, and its decline in advanced fibrosis contributes to disease progression through ineffective immune surveillance.

### 3.2. STING Signaling Regulates NK Cell Dysfunction Across Liver Fibrosis Stages in MASH

**Figure 2:**
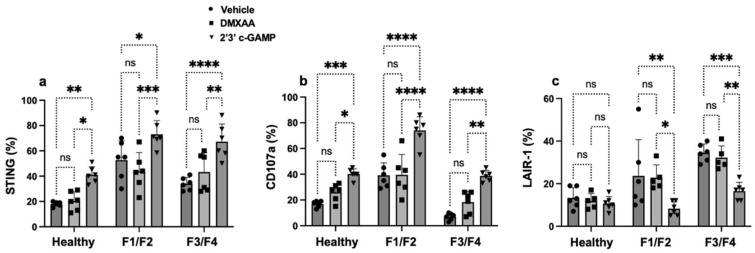
STING signaling orchestrates NK Cell impairment along liver fibrosis progression in MASH. Following isolation, NK cells from healthy individuals and MASH patients with early (F1/F2) and advanced (F3/F4) stages of liver fibrosis were seeded at a concentration of 1 × 10^6^ cells/mL. The cells were then treated for 24 h with STING agonists 5,6-dimethylxanthenone-4-acetic acid (DMXAA; D5817, MERCK) or 2′3′-cyclic GMP-AMP (2′3′-cGAMP; tlrl-nacga23-02, InvivoGen) each at a final concentration of 10 µM. DMSO was used as the vehicle control. The expression levels of (**a**) CD107a, (**b**) NKp46, and (**c**) LAIR-1 on NK cells were assessed by flow cytometry. Isotype-matched IgG antibodies conjugated with appropriate fluorochromes were used as controls in the flow cytometry assays. Data are presented as the mean ± standard deviation (SD). Statistical significance was determined using Newman–Keuls two-way ANOVA with the following notations: * *p* < 0.01, ** *p* < 0.001, *** *p* < 0.0001, and **** *p* < 0.00001. ns = not significant. Group sizes included healthy controls (*n* = 5), patients with early fibrosis (*n* = 5), and patients with advanced fibrosis (*n* = 5).

Based on our results, we further explored the impact of STING expressions on NK cell activity in an attempt to restore their function, we have used STING agonists known to modulate the protein expressions via changes in its translocation and configuration [19]; DMXAA (murine-specific) and 2′3′-cyclic GMP-AMP (2′3′-cGAMP, the endogenous human ligand). NK cells were isolated from healthy controls, F1/F2, and F3/F4 scores, and treated with STING agonists for 24 h before assessment for NK cells inhibitory and activatory receptors and STING expressions as mentioned in the Materials and Methods. Figure 2a showed that STING protein expression was significantly upregulated following 2′3′-cGAMP stimulation, but not after treatment with DMXAA in all tested NK cells. Healthy obtained NK cells showed increased STING expression from 18% ± 3.2 in the vehicle-treated cells to 40% ± 6.8 following 2′3′-cGAMP exposure (2.2 fold, *p* < 0.01), while no changes were significantly observed with DMXAA. Moreover, NK cells from F1/F2 MASH patients showed elevated STING expression from 52% ± 13.2 in the vehicle group to 66% ± 6 following 2′3′-cGAMP stimulation (1.3 fold, *p* < 0.001), with no detectable alteration following DMXAA treatment. Similarly, NK cells from F3/F4 MASH patients exhibited a significant noticeable increase in STING expression from 25.1% ± 1.2 vehicle-treated cells to 63% ± 13.8 following 2′3′-cGAMP exposure (2.5 fold, *p* < 0.01).

To correlate the modulation in STING expression with changes in NK cells activity, we have assessed the CD107a expression. Figure 2b demonstrated that stimulation with 2′3′-cGAMP significantly enhanced CD107a surface expression across all groups, while DMXAA showed no measurable effect. CD107a expression modestly increased in NK cells by 2, 1.8, and 4.75 fold following 2′3′-cGAMP treatment, in healthy and F1/F2 and F3/F4, respectively (*p* < 0.01). DMXAA-treated cells showed no significant changes in all tested groups.

To further determine whether NK cells inhibitory receptors are modulated following the use of STING agonists, LAIR-1 expressions were evaluated via flow cytometry. Figure 2c revealed that stimulation with 2′3′-cGAMP significantly reduced LAIR-1 expression in NK cells from F1/F2 scores by 1.53 fold (*p* < 0.01). A similar trend was observed in NK cells from the F3/F4 scores, where LAIR-1 expression declined by 1.85 fold following 2′3′-cGAMP stimulation. DMXAA treatment did not significantly alter LAIR-1 expression in NK cells from either F1/F2 or F3/F4 MASH patients (*p* = ns). Moreover, both agonists failed to modulate LAIR-1 expression in NK cells derived from healthy individuals.

These findings demonstrate that the human STING agonist 2′3′-cGAMP, but not the murine-specific DMXAA, effectively restores STING protein expression and enhances NK cell activity across all disease stages, including advanced fibrosis. In addition, our results suggest that pharmacologic activation of STING using 2′3′-cGAMP may overcome the post-transcriptional suppression observed in fibrotic NK cells, offering a promising strategy to reinvigorate their cytotoxic potential in liver fibrosis.

### 3.3. Insulin Signaling Promotes STING Expression and Restores NK Cell Function in Patients with MASH

**Figure 3:**
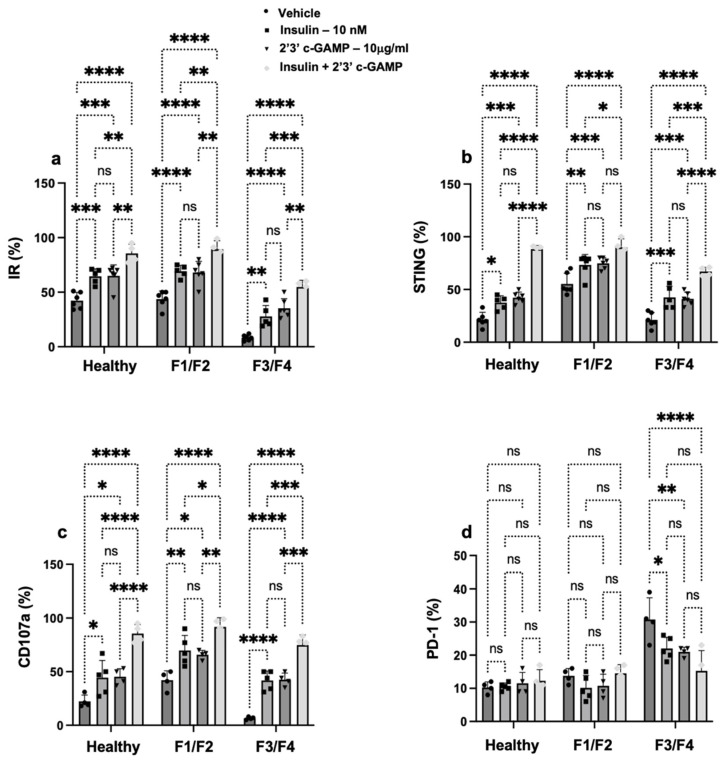
Insulin signaling enhances STING expression and restores NK Cell function in MASH patients. Following isolation, NK cells from healthy controls and MASH patients with early (F1/F2) and advanced (F3/F4) fibrosis scores were seeded at 1 × 10^6^ cells/mL and treated with the STING agonists 2′3′-cyclic GMP-AMP (2′3′-cGAMP; tlrl-nacga23-02, InvivoGen, California, USA) at a final concentration of 10 µM or recombinant human insulin (91077C, MERCK, Jerusalem, Israel), or both for 24 h. DMSO is used as a vehicle. The percentages of cells expressing (**a**) insulin receptor (IR), (**b**) STING, (**c**) CD107a, and (**d**) PD-1in NK cells were measured by flow cytometry. In the flow cytometry experiments, an isotype IgG antibody labeled with the relevant fluorochrome was used as a control. The data are presented as the average ± SD. Significance was determined using Newman–Keuls two-way analysis of variance (ANOVA), * *p* < 0.01, ** *p* < 0.001, *** *p* < 0.0001, and **** *p* < 0.00001. ns = not significant. Healthy (*n* = 5), early liver fibrosis (*n* = 5), and advanced liver fibrosis (*n* = 5).

In a previous study, we have shown that insulin receptors on NK cells have been shown to modulate their metabolism, cytotoxicity, and cytokine production [22]. However, it remains poorly understood whether STING pathway dysregulation in NK cells alters their insulin responsiveness and how this might influence NK cell functionality across stages of liver fibrosis. As mentioned in the Materials and Methods, the 2′3′-cGAMP STING agonist was assessed for its effects on modulating NK cell IR. Insulin treatment on NK cells was used as a positive control. Figure 3a shows that healthy NK cells treated with insulin significantly increased IR expression from 47% ± 4.1 in the vehicle-treated cells to 60% ± 2.8 (1.27 fold, *p* < 0.0001). Comparable results were obtained following 2′3′-cGAMP with a 1.3-fold increase (*p* < 0.01). Notably, co-treatment with both insulin and 2′3′-cGAMP resulted in a synergistic increase in IR expression and reached 91% ± 4.2.

Next, we assessed whether elevated expressions of IR on NK cells are associated with changes in STING expression and, consequently, could affect NK cell activity. Figure 3b demonstrates an elevated expression of STING in NK cells following insulin treatment in healthy and MASH NK cells of F1/F2 and F3/F4 by 2, 1.26, and 1.9 fold, respectively. In parallel, a similar fold increase in STING expression was noticed following 2′3′-cGAMP treatments in all tested groups, and the data were statistically significant. Combined therapy of both insulin and 2′3′-cGAMP caused a 3.6, 1.5, and 2.7 fold increase in STING expression in the healthy and F1/F2 and F3/F4 NK cells, respectively (*p* < 0.01).

To assess whether the upregulation of both IR and STING expression is translated into improved NK cell cytotoxicity, we evaluated CD107a expression. Figure 3c illustrates elevated expressions of CD107a in NK cells following insulin treatment in healthy and MASH NK cells of F1/F2 and F3/F4 by 1.9, 1.7, and 4.7 fold, respectively, compared to each group’s vehicle treatment NK cells. Similarly, an increase in CD107a expression was observed following the 2′3′-cGAMP treatments in all tested groups, and the data were statistically significant. Combined therapy of both insulin and 2′3′-cGAMP caused a 3.0, 2.1-fold increase in the healthy and F1/F2, respectively (*p* < 0.01) and, surprisingly, a 6.6-fold increase in CD107a expression in the F3/F4-scored NK cells as compared to vehicle treatment NK cells.

To evaluate immune exhaustion in the context of liver fibrosis, we assessed PD-1 expression levels in peripheral NK cells across healthy controls, early-stage fibrosis (F1/F2), and advanced fibrosis (F3/F4; Figure 3d). In healthy individuals, PD-1 expression was low (13%). Similarly, PD-1 levels in the F1/F2 group remained comparable to those of the healthy controls (fold change < 1.2, *p* = not significant compared to healthy). In contrast, patients with advanced fibrosis (F3/F4) exhibited a marked upregulation of PD-1 expression, with levels reaching up to 35%, representing a 3-fold increase compared to healthy controls. Treatment with insulin or the STING agonist cGAMP alone significantly reduced PD-1 expression levels in NK cells from patients with advanced liver fibrosis. When administered in combination, insulin and cGAMP had an additive effect, leading to a more pronounced reduction in PD-1 expression (*p* < 0.01), effectively reversing the exhaustion phenotype.

Our findings demonstrate that STING and insulin signaling pathways converge to regulate NK cell function, with co-activation significantly enhancing insulin receptor and STING expression and restoring cytotoxic activity, particularly in NK cells from MASH patients with advanced fibrosis of F3/F4. These results suggest that while insulin could directly impact STING immune expressions, synergistic therapeutic potential for NK cell dysfunction in metabolically impaired fibrotic environments is achieved via both insulin and STING pathways.

## 4. Discussion

Several checkpoints have recently emerged in modulating disease progressions in MASH patients and a few of them focused on the immune impact of the disease [22,23,24,25,26]. NK cells play a major part in delaying liver fibrosis while many studies demonstrate that they lose their antifibrotic effects in advanced liver diseases [20,21]. We have previously showed insulin signaling as a potential natural killer cell checkpoint in fatty liver disease [22]. Serum insulin, acting through IR, directly enhanced cytotoxic NK activity toward HSCs, alleviating fibrosis in early hyper-insulinemic stages of insulin resistance. Reduced serum insulin levels and/or reduced expression of IR on NK cells prevented NK cell activation and induced their impairment by apoptosis in advanced liver injury stages [22]. In our current study, we have introduced an immune-metabolic checkpoint traditionally known for its involvement in antiviral immunity, the STING pathway which has recently been identified as a key regulator of liver inflammation and fibrosis1-3. Elevated STING expression has been observed in monocyte-derived macrophages [7,8,9,10,11]. Our data confirmed an inhibited expressions in STING on NK cells from MASH patients with advanced liver fibrosis of Metavir scores of F3 and F4. STING expressions positively correlated with NK cells activity and inversely associated with exhaustion markers of PD-1, TIGIT and LAG-3. Moreover, our findings underscore a synergistic relationship wherein insulin and STING pathways restore NK cell cytotoxicity, particularly in advanced fibrotic stages. Notably, the STING agonist 2′3′-cGAMP similarly upregulates IR expression, and their combined application results in a synergistic increase, suggesting a crosstalk between metabolic and innate immune signaling pathways. Insulin and 2′3′-cGAMP treatments not only augmented STING expression in NK cells but also significantly enhanced their cytotoxic activity, as evidenced by increased CD107a expression. This enhancement was most pronounced in NK cells from patients with advanced fibrosis (F3/F4). Furthermore, the restoration of NK cell activity has implications for HSC regulation. Activated NK cells are known to induce apoptosis in activated HSCs, thereby mitigating fibrosis progression. Our findings suggest enhancing NK cell function through insulin and STING pathway modulation could potentiate this antifibrotic mechanism. In conclusion, the convergence of insulin and STING signaling pathways offers a promising therapeutic avenue to restore NK cell function and counteract liver fibrosis in MASH. Future research should investigate the underlying mechanisms of this interaction and evaluate the potential of targeting these pathways as a therapeutic strategy for managing liver fibrosis.

## Figures and Tables

**Table 1:** Patient demographic and clinical characteristics. Demographic and clinical parameters of study participants grouped by liver fibrosis stage: healthy controls (*n* = 10), patients with early liver fibrosis (F1/F2, *n* = 10), and patients with advanced liver fibrosis (F3/F4, *n* = 10). Continuous variables are presented as the mean ± standard deviation (SD); categorical variables are presented as number and percentage (%). *p*-values were calculated using ANOVA for continuous variables and the chi-square test for categorical variables. *p* < 0.05 was considered statistically significant.

Variable	Healthy (*n* = 10)	F1/F2 (*n* = 10)	F3/F4 (*n* = 10)	*p*-Value
Age, Years	36.5 ± 8.2	39.3 ± 9.1	38.10 ± 9.6	ns
Male, *n* (%)	6 (60%)	5 (50%)	4 (40%)	ns
Male, *n* (%)	4 (40%)	5 (50%)	6 (60%)	ns
Fibroscan CAP (dB/m)	210 ± 12	258 ± 13	359.5 ± 16	<0.01
ALT, U/L	22 ± 7	29 ± 7	59 ± 18	<0.001
AST, U/L	29 ± 5	39 ± 10	49 ± 17	<0.001

**Table 2:** Expression of exhaustion markers on peripheral NK Cells across Fibrosis Stages. The percentage of NK cells expressing PD-1, TIGIT, and LAG-3 was assessed in peripheral blood samples from healthy donors and those with liver fibrosis, using flow cytometry. The data are presented as the average ± SD. Significance was determined using Newman–Keuls two-way analysis of variance (ANOVA), * *p* < 0.01 compared to healthy donors, healthy (*n* = 10), early liver fibrosis (*n* = 10), and advanced liver fibrosis (*n* = 10).

NK Donor	PD-1 (%)	TIGIT (%)	LAG-3 (%)
Healthy	8 ± 2.5	13 ± 1.4	9 ± 1.5
F1/F2	11 ± 3.3	15 ± 3.2	8 ± 1.5
F3/F4	32 ± 4.7 *	28 ± 5.3 *	21 ± 4.9 *

## Data Availability

This study’s original findings and contributions are detailed in this article itself. For any additional inquiries or information, it is recommended to contact the corresponding authors of this study. They will be able to provide further clarification and address any specific questions related to this research.

## Supplementary Materials

The following supporting information can be downloaded at: https://www.mdpi.com/article/10.3390/cells14130941/s1. Figure S1. The gating strategy of flow cytometry plots for peripheral NK cell analysis. (A) Represents the NK cell population following isolation, gated based on forward scatter area (FSC-A) and side scatter area (SSC-A) to identify viable cells. (B) Gate 1 is set to identify the isolated NK cells following the Human NK Cell Isolation Kit and their purity was defined as CD56^+^ CD3^−^ cells to effectively exclude NKT cells from the analysis. (C–E) are derived from Gate 1 and further characterize the NK cell population expressed NKp46 in (C) Healthy, (D) F1/F2, and (E) F3/F4 donors. Figure S2. NK cells immunophenotyping. The mean fluorescence intensity (MFI) of cells expressing (a) NKp46 and (b) LAIR-1, in NK from healthy controls and metabolic dysfunction-associated steatohepatitis (MASH) patients with early (F1/F2) and advanced (F3/F4) fibrosis scores as determined by METAVIR, was measured by flow cytometry. (The data are presented as the average ± SD. Significance was determined using Newman-Keuls two-way analysis of variance (ANOVA), * *p* < 0.01, ** *p* < 0.001, *** *p* < 0.0001, and **** *p* < 0.00001. Healthy (n = 10), early liver fibrosis (n = 10), and advanced liver fibrosis (n = 10).
