# Supplementary material for: Insulin Modulates NK Cell Activity in Liver Fibrosis MASH Patients via the STING Pathway
Source: Cells. 2025 Jun 20;14(13):941. doi: 10.3390/cells14130941 (PMC12249237; doi:10.3390/cells14130941)
Supplement: Supplementary file 1 [file cells-14-00941-s001.zip › cells-3654479-supplementary.pdf]

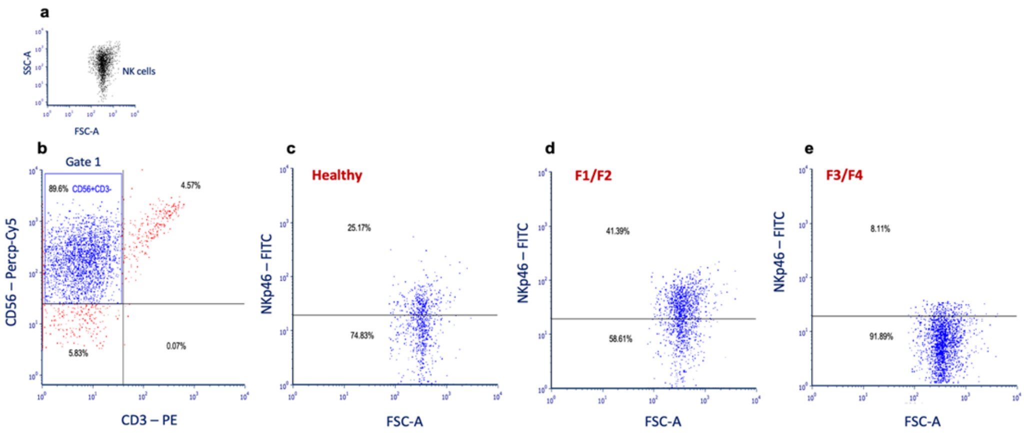

Figure S1. The gating strategy of flow cytometry plots for peripheral NK cell analysis. (A) Represents the NK cell population following isolation, gated based on forward scatter area (FSC-A) and side scatter area (SSC-A) to identify viable cells. (B) Gate 1 is set to identify the isolated NK cells following the Human NK Cell Isolation Kit and their purity was defined as $CD56^{+}$ $CD3^{-}$ cells to effectively exclude NKT cells from the analysis. (C-E) are derived from Gate 1 and further characterize the NK cell population expressed NKp46 in (C) Healthy, (D) F1/F2, and (E) F3/F4 donors.

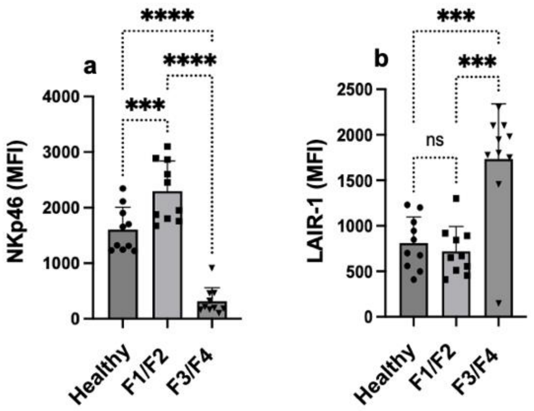

Figure S2. NK cells immunophenotyping. The mean fluorescence intensity (MFI) of cells expressing (a) NKp46 and (b) LAIR-1, in NK from healthy controls and metabolic dysfunction-associated steatohepatitis (MASH) patients with early (F1/F2) and advanced (F3/F4) fibrosis scores as determined by METAVIR, was measured by flow cytometry. (The data are presented as the average ± SD. Significance was determined using Newman-Keuls two-way analysis of variance (ANOVA), *$p < 0.01$, **$p < 0.001$, ***$p < 0.0001$, and ****$p < 0.00001$. Healthy (n=10), early liver fibrosis (n=10), and advanced liver fibrosis (n=10).
